# Baseline treatments and metabolic control of 288,913 type 2 diabetes patients in a 10-year retrospective cohort in Malaysia

**DOI:** 10.1038/s41598-023-44564-y

**Published:** 2023-10-13

**Authors:** Kim Sui Wan, Feisul Mustapha, Arunah Chandran, Shubash Shander Ganapathy, Nurhaliza Zakariah, Sivarajan Ramasamy, Gunenthira Rao Subbarao, Muhammad Fadhli Mohd Yusoff

**Affiliations:** 1 Institute for Public Health, National Institutes of Health, Ministry of Health Malaysia, Setia Alam, 40170 Shah Alam, Selangor Malaysia; 2 Disease Control Division, Ministry of Health Malaysia, Federal Government Administration Centre, 62590 Putrajaya, Malaysia; 3 State Health Department of Negeri Sembilan, Ministry of Health Malaysia, Jalan Rasah, 70300 Seremban, Negeri Sembilan Malaysia; 4 Medical Development Division, Ministry of Health Malaysia, Federal Government Administration Centre, 62590 Putrajaya, Malaysia

**Keywords:** Endocrinology, Endocrine system and metabolic diseases, Endocrine system and metabolic diseases

## Abstract

Diabetes is one of the quickest-growing global health emergencies of the twenty-first century, and data-driven care can improve the quality of diabetes management. We aimed to describe the formation of a 10-year retrospective open cohort of type 2 diabetes patients in Malaysia. We also described the baseline treatment profiles and HbA1c, blood pressure, and lipid control to assess the quality of diabetes care. We used 10 years of cross-sectional audit datasets from the National Diabetes Registry and merged 288,913 patients with the same identifying information into a 10-year open cohort dataset. Treatment targets for HbA1c, blood pressure, LDL-cholesterol, HDL-cholesterol, and triglycerides were based on Malaysian clinical practice guidelines. IBM SPSS Statistics version 23.0 was used, and frequencies and percentages with 95% confidence intervals were reported. In total, 288,913 patients were included, with 62.3% women and 54.1% younger adults. The commonest diabetes treatment modality was oral hypoglycaemic agents (75.9%). Meanwhile, 19.3% of patients had ≥ 3 antihypertensive agents, and 71.2% were on lipid-lowering drugs. Metformin (86.1%), angiotensin-converting enzyme inhibitors (49.6%), and statins (69.2%) were the most prescribed antidiabetic, antihypertensive, and lipid-lowering medications, respectively. The mean HbA1c was 7.96 ± 2.11, and 31.2% had HbA1c > 8.5%. Only 35.8% and 35.2% attained blood pressure < 140/80 mmHg and LDL-cholesterol < 2.6 mmol/L, respectively. About 57.5% and 52.9% achieved their respective triglyceride and HDL-cholesterol goals. In conclusion, data integration is a feasible method in this diabetes registry. HbA1c, blood pressure, and lipids are not optimally controlled, and these findings can be capitalized as a guideline by clinicians, programme managers, and health policymakers to improve the quality of diabetes care and prevent long-term complications in Malaysia.

## Introduction

The International Diabetes Federation cautions that diabetes is one of the quickest-growing global health emergencies of the twenty-first century^1^. The worldwide diabetes prevalence among populations aged 20–79 years in 2021 was estimated to be 10.5%, with over half a billion people living with this non-communicable disease^2^. Malaysia, a country in the Southeast Asian region, has a diabetes prevalence of around 20.0%, almost double the global average prevalence^1^. The sheer disease burden of diabetes and its complications makes this disease a key priority area in the Malaysian nation-building plan^3^.

The Lancet Commission on Diabetes emphasises the importance of delivering data-driven care to improve the quality of diabetes management to transform patient lives^4^. Diabetes registers involve structured data collection that serves multiple purposes^4^. On a patient level, healthcare practitioners can use data to provide feedback and individualised management. Additionally, data can be utilised on a system level to identify care gaps and benchmark performance^5^. Policy-wise, diabetes registers can be used as a surveillance tool to monitor disease patterns and burdens^5^. Given the lack of large longitudinal cohort studies to monitor trends meaningfully, administrative data can contribute crucially to informing clinical and public health practice^4^.

The Ministry of Health (MOH) Malaysia established the National Diabetes Registry in 2009 to track diabetes control and clinical outcomes of patients managed at MOH health clinics^6^. The registry also aims to enable research to be carried out to improve the quality of care provided to patients^6^. Annual diabetes clinical audits are conducted as part of the Quality Assurance Programme, using glycosylated haemoglobin A1c (HbA1c) control as the proxy of the quality of care^7^. These audits involve systematic data entry by healthcare providers into the National Diabetes Registry database^6^.

While the registry is crucial to capture essential clinical information, the annual clinical audits are cross-sectional in design^6^. The primary issue with the database is its inability to track individual patients over time^8^. In other words, no specific cohort of patients is followed up, making longitudinal analysis impossible by default^8^. The unavailability of panel data presented a significant methodological gap because many research questions could not be answered. Therefore, this paper aims to describe the formation of a 10-year retrospective open cohort of type 2 diabetes patients in Malaysia. We also describe the baseline treatment profiles and HbA1c, blood pressure (BP), and lipid control to assess the quality of diabetes care.

## Methods

### Study design

This was a 10-year retrospective open cohort study from 2011 to 2020. Cohort design gives a higher quality of evidence than other observational study designs, such as cross-sectional study, because the temporal relationship between exposure and outcome is clear^9^. Retrospective, rather than prospective cohort, was chosen because a prospective cohort study would be expensive, time-consuming, and labour-intensive^9^. Open instead of closed cohort was used because it mirrored the real-world scenario with dynamic memberships^9^. Furthermore, the open cohort design yielded a larger sample size, which increased the statistical power^9^.

### Study population

The study population was patients with type 2 diabetes (T2D) treated in all public health clinics in Malaysia. Over 80% of diabetes patients in Malaysia received their treatments from public healthcare facilities^10^. The inclusion criteria for this cohort were patients with T2D, adults aged 18 years and above, and had been randomly selected for at least two clinical audits between 2011 and 2020. A minimum of two audit data was needed as a longitudinal study required data from at least two separate time points. Patients with all other types of diabetes, such as type 1 diabetes, gestational diabetes, and prediabetes, were excluded.

### Data integration

The data source came from the Malaysian National Diabetes Registry. The registry is a web-based system that contains two components: the ‘registry’ dataset and the ‘clinical audit’ dataset^6^. The registry dataset included patients with T2D, type 1 diabetes, and other types of diabetes from hospital and health clinic settings. This dataset contained basic demographic information. The clinical audit dataset was formed every year when a subset of T2D patients on active follow-up in public health clinics was audited as part of the Quality Assurance Programme in the MOH^6,7^. The patients to be audited each year were randomly selected by the diabetes program managers centrally, and healthcare professionals in health clinics would then manually transcribe the required audit information of the selected patients into the web-based registry. All patients had the same probability of being selected, regardless of whether they had been audited^6^. Patients would usually have several visits to the health clinics each year. Their last observed clinical information was used in the audits to represent the year’s performance. Unlike the registry dataset, the audit dataset had more information as it captured clinical variables, pharmacological treatment, and clinical care outcomes^6^.

**Figure 1:**
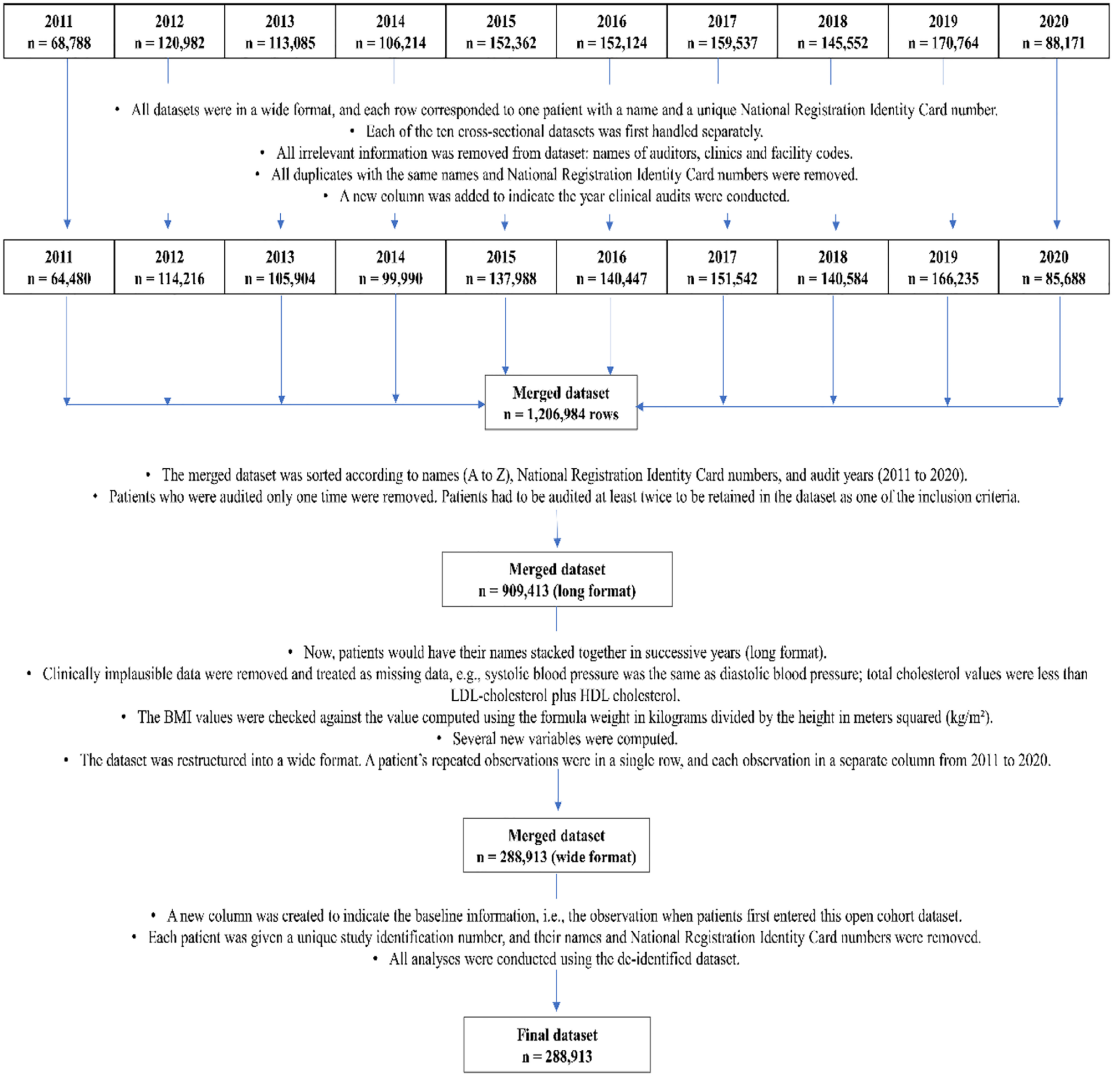
Data integration to form the study cohort dataset.

We merged a 10-year retrospective open cohort using the data integration method. Data integration (also called data fusion, data matching, data merging, or data linkage) methodology is an emerging field that enables researchers to pool data drawn from multiple existing studies^11^. At the fundamental level, combining data means merging information from different datasets with some common variables^11^. Data linkage is one of the big data techniques that has been applied in research settings for diabetes to generate valuable and actionable insights^12^. Fig. 1 outlines the steps implemented to form this cohort dataset. An initial 1,277,579 audit data was managed to form a 10-year retrospective open cohort with 288,913 unique patients with at least two data points across the 10-year period.

### Study variables

The baseline variables, such as demography, comorbidities, complications, treatment profiles, and metabolic control, were captured when patients entered the cohort. The demography comprised of age (< 60 and ≥ 60 years), sex (men and women), and the major ethnicities in Malaysia (Malay, Chinese, Indian, and Others). Older adults were patients aged ≥ 60 years based on the United Nations definition^13^. Young-onset T2D was a diagnosis made in individuals aged < 40 years^14^. The duration of diabetes was < 5, 5–10, and > 10 years.

The comorbidities were overweight, obese, abdominal obesity, dyslipidaemia, and hypertension. We adapted the Malaysian clinical practice guidelines (CPG) to define overweight (body mass index, BMI 23.0–27.4 kg/m^2^), obese I (27.5–34.9 kg/m^2^), obese II (35.0–39.9 kg/m^2^), and obese III (≥ 40.0 kg/m^2^)^15^. The lower cut-offs were consistent with the World Health Organization (WHO) recommendations for Asian populations because Asian populations had higher risks of cardiovascular diseases at BMIs lower than the standard WHO international classifications^16^. Abdominal obesity was defined as having waist circumference ≥ 90 cm for men and ≥ 80 cm for women^15^. Meanwhile, dyslipidaemia and hypertension were based on clinical diagnoses by their treating physicians or patients using lipid-lowering or antihypertensive agents, respectively. Diabetes complications, namely ischemic heart disease, stroke, retinopathy, nephropathy, foot ulcers, and non-traumatic lower extremity amputation, were based on clinical diagnoses.

The treatment profiles included diabetes treatment modality, antihypertensive, lipid-lowering, and antiplatelet agents. Diabetes treatment modality comprised of lifestyle modification without pharmacological agents, oral hypoglycaemic agents (OHA) only, insulin only, and both OHA and insulin. The types of OHAs were biguanides (metformin), sulphonylureas, alpha-glucosidase inhibitors, meglitinides, glitazones, and others. The types of antihypertensive drugs included angiotensin-converting enzyme (ACE) inhibitors, angiotensin receptor blockers (ARB), beta-blockers, calcium channel blockers, diuretics, peripheral alpha-blockers, centrally acting agents, and others. Acetylsalicylic acid (aspirin), ticlopidine, and others made up the antiplatelet drugs, while statins, fibrates, and others were lipid-lowering drugs. Polypharmacy was the use of five or more types of pharmacological agents^17^.

The metabolic control comprised HbA1c, BP, and lipid control. The HbA1c cut-offs were ≤ 6.5%, < 7.0%, and > 8.5%. The proportion of T2D patients with HbA1c ≤ 6.5% is a quality indicator under the Quality Assurance Programme, and the target is ≥ 30.0%^7^. HbA1c < 7.0% is a more suitable target for most T2D patients based on local CPG and the International Diabetes Federation (IDF) recommendations^18,19^. The proportion of T2D patients with HbA1c > 8.5% is a Key Performance Index (KPI) indicator, and the target is < 20% for primary care clinics in Malaysia^18^. The BP < 140/80 mmHg was adopted from the local and IDF recommendations^18,19^. We also used the common BP cut-off of < 130/80 mmHg, as reported in a meta-analysis^20^. LDL-cholesterol is the primary lipid target, while triglycerides, HDL-cholesterol, and non-HDL-cholesterol are the secondary targets^18,21^. The LDL-cholesterol < 2.6 mmol/L, triglycerides < 1.7 mmol/L, HDL-cholesterol > 1.0 mmol/L for men and > 1.3 mmol/L for women, and non-HDL-cholesterol < 3.4 mmol/L are consistent with the Malaysian and European CPGs^18,21^.

### Statistical analysis

IBM SPSS Statistics version 23.0 was used for analysis. For normally distributed data, variables were presented in mean ± standard deviation (SD), while median and inter-quartile range (IQR) were presented for skewed data. Both frequency and percentages were reported for categorical variables. The 95% confidence intervals were also presented for the metabolic control.

### Ethical approval

The ethics approval was given by the Medical Research and Ethics Committee under the MOH Malaysia (NMRR-ID-23-00396-REO). All methods followed the Declaration of Helsinki and the Malaysian Good Clinical Practice Guidelines. The Medical Research and Ethics Committee waived the need for informed consent as the registry was analysed retrospectively using secondary datasets.

## Results

**Figure 2:**
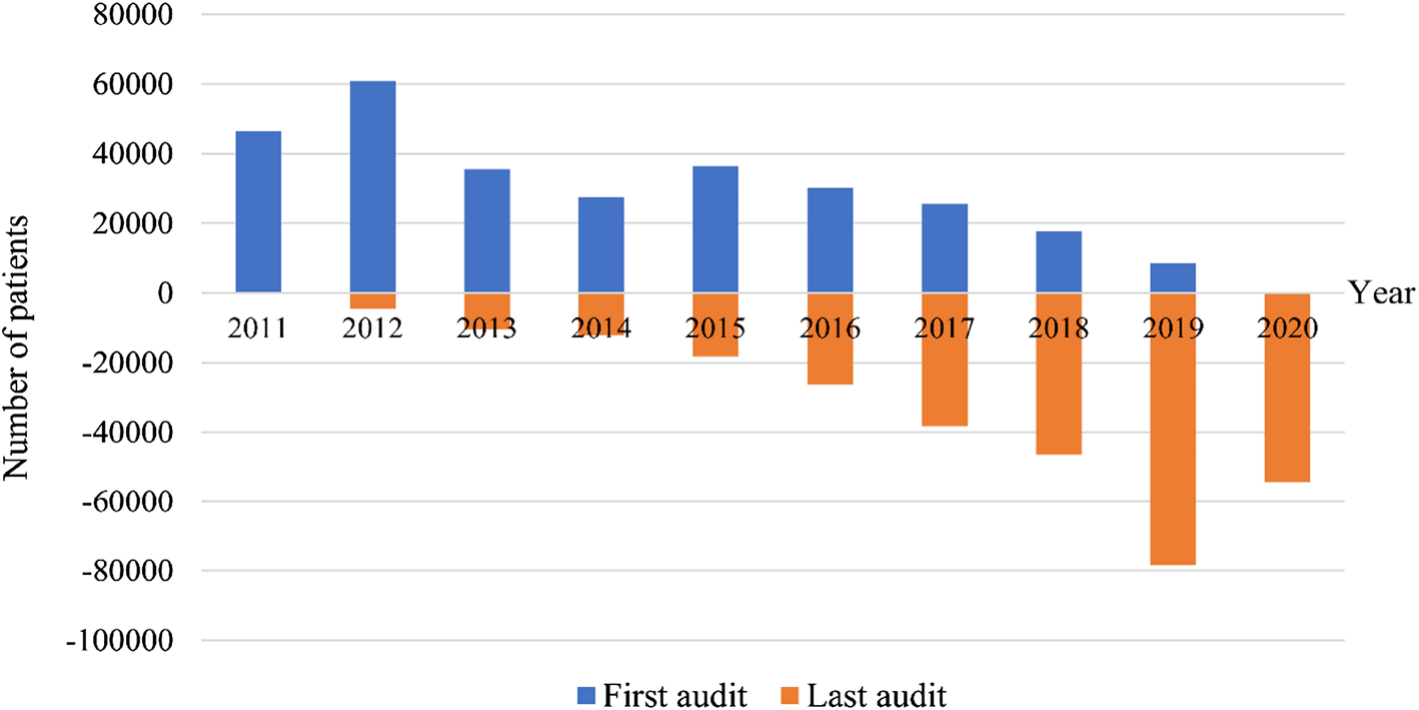
Distribution of first and last audits by chronological years.

A total of 288,913 eligible patients were included in this 10-year open cohort. The total person-years of follow-ups were 1,078,101 years, with a mean of 3.7 ± 2.3 person-years. The range of follow-ups was from 2 to 9 years. Figure 2 shows the first and last audit data distribution by chronological years. The median years of the first and last audits were 2014 and 2018, respectively.

Table 1 provides the baseline characteristics captured when they had their first clinical audit. There were more younger adults below 60 years, women, and Malay ethnicity, and most patients were diagnosed with diabetes for less than 5 years. It was observed that 31,898 or 11.0% of patients had young-onset T2D at below 40 years. A staggering 84.5% of patients were overweight or obese based on the BMI classification, while 78.5% had abdominal obesity. Meanwhile, hypertension and dyslipidaemia were observed in 81.6% and 76.1% of patients, respectively. Between 0.4 and 7.2% of our patients had diabetes-related complications. Including T2D, 93.4% of our patients had multimorbidity, which is the co-occurrence of two or more chronic conditions.

**Table 1:** Baseline characteristics of the cohort, n = 288,913.

Characteristics		n (%)
Age, years	Mean ± standard deviation	58.7 ± 10.8
	< 60	156,405 (54.1)
	≥ 60	132,508 (45.9)
Age at onset, years	Mean ± standard deviation	53.1 ± 10.8
	< 40	31,898 (11.0)
	40–59	182,328 (63.1)
	≥ 60	74,687 (25.9)
Duration of diabetes, years	Median (inter-quartile range)	4.1 (5.9)
	< 5	161,977 (56.1)
	5–10	80,689 (27.9)
	> 10	46,247 (16.0)
Sex	Men	108,937 (37.7)
	Women	179,976 (62.3)
Ethnicity	Malay	192,681 (66.7)
	Chinese	46,553 (16.1)
	Indian	27,138 (9.4)
	Others	22,541 (7.8)
BMI category (n = 284,141)	Mean ± standard deviation, kg/m^2^	27.7 ± 5.1
	Underweight (< 18.5 kg/m^2^)	4,140 (1.5)
	Normal (18.5 to 22.9 kg/m^2^)	39,821 (14.0)
	Overweight (23.0 to 27.4 kg/m^2^)	107,659 (37.9)
	Obese I (≥ 27.5 to 34.9 kg/m^2^)	107,707 (37.9)
	Obese II (≥ 35.0 to 39.9 kg/m^2^)	17,898 (6.3)
	Obese III (≥ 40.0 kg/m^2^)	6,916 (2.4)
Waist circumference, cm (n = 263,703)	Overall, mean ± standard deviation	92.1 ± 12.2
	Men WC, mean ± standard deviation	94.2 ± 12.2
	Women WC, mean ± standard deviation	90.9 ± 12.1
Abdominal obesity (n = 263,703)	Overall	206,929 (78.5)
	Male, WC ≥ 90 cm	65,949 (66.5)
	Female, WC ≥ 80 cm	140,980 (85.7)
Hypertension	Yes	235,835 (81.6)
Dyslipidaemia	Yes	219,910 (76.1)
Ischemic heart disease	Yes	11,286 (3.9)
Stroke	Yes	2,990 (1.0)
Retinopathy	Yes	18,357 (6.4)
Nephropathy	Yes	20,763 (7.2)
Foot ulcer	Yes	2470 (0.9)
Non-traumatic lower extremity amputation	Yes	1191 (0.4)
Number of comorbidities and complications	None	19,457 (6.7)
	One	73,016 (25.3)
	Two	157,940 (54.7)
	Three	31,351 (10.9)
	Four	6071 (2.1)
	Five or more	1078 (0.4)

Missing data—Body mass index: 4772 (1.6%); waist circumference and abdominal obesity: 25,210 (8.7%).

Table 2 presents the patients’ baseline treatment profiles, while Table 3 demonstrates their metabolic control. Metformin was the most common drug (86.1%), and treatment with ‘oral hypoglycaemic agents only’ was the most common diabetes treatment modality. Meanwhile, one in five patients was treated with insulin. The mean HbA1c was 7.97%, and 31.9% and 40.9% of patients achieved HbA1c < 6.5% and < 7.0%, respectively. Notably, 31.2% of patients had HbA1c > 8.5%.

**Table 2:** Treatment profiles, n = 288,913.

Characteristics		n (%)
Antidiabetic medications	Biguanides (metformin)	248,641 (86.1)
	Sulphonylureas	153,663 (53.2)
	Alpha-glucosidase inhibitor	11,589 (4.0)
	Meglitinides	350 (0.1)
	Glitazones	2775 (1.0)
	Other oral hypoglycaemic agents	3365 (1.2)
	Insulin	58,597 (20.2)
Diabetes treatment modality	Lifestyle modification only	11,127 (3.9)
	Oral hypoglycaemic agent (OHA) only	219,189 (75.9)
	Insulin only	12,564 (4.3)
	Both OHA and insulin	46,033 (15.9)
Antihypertensive drugs	Angiotensin-converting enzyme (ACE) inhibitor	143,390 (49.6)
	Angiotensin receptor blocker (ARB)	14,763 (5.1)
	Beta-blocker	67,776 (23.5)
	Calcium channel blocker	129,256 (44.7)
	Diuretic	68,008 (23.5)
	Peripheral alpha-blocker	9169 (3.2)
	Centrally acting agent	686 (0.2)
	Other antihypertensive drugs	21 (0.0)
Number of antihypertensive drugs	None	60,741 (21.0)
	One	92,332 (32.0)
	Two	80,208 (27.7)
	Three	43,282 (15.0)
	More than three	12,350 (4.3)
Lipid-lowering drugs	Statin	200,029 (69.2)
	Fibrate	8748 (3.0)
	Other lipid-lowering drugs	1728 (0.8)
Number of lipid-lowering drugs	None	83,259 (28.8)
	One	201,414 (69.7)
	Two or more	4240 (1.5)
Antiplatelet drugs	Acetylsalicylic acid (aspirin)	65,321 (22.6)
	Ticlopidine	2709 (0.9)
	Other antiplatelet drugs	1155 (0.7)
Number of antiplatelet drugs	None	220,856 (76.4)
	One	67,812 (23.5)
	Two	245 (0.1)
Polypharmacy	Five or more types of drugs	115,814 (40.1)

**Table 3:** Metabolic control, n = 288,913.

Characteristics		n (%)	95% CI
HbA1c, % (n = 284,106)	Mean ± SD	7.96 ± 2.11	7.95–7.97
	≤ 6.5%	90,644 (31.9)	31.7–32.1
	< 7.0%	116,327 (40.9)	40.8–41.1
	> 8.5%	88,686 (31.2)	31.1–31.4
Systolic blood pressure, mmHg (n = 288,733)	Mean ± SD	135.3 ± 17.3	135.2–135.3
	< 140 mmHg	176,185 (61.0)	60.8–61.2
	< 130 mmHg	101,165 (35.0)	34.9–35.2
Diastolic blood pressure, mmHg (n = 288,731)	Mean ± SD	78.3 ± 10.0	78.2–78.3
	< 80 mmHg	139,592 (48.3)	48.2–48.5
Blood pressure, mmHg (n = 288,730)	< 140/80 mmHg	103,238 (35.8)	35.6–35.9
	< 130/80 mmHg	70,226 (24.3)	24.2–24.5
Total cholesterol, mmol/L (n = 283,033)	Mean ± SD	5.13 ± 1.23	5.13–5.13
LDL-cholesterol, mmol/L (n = 244,642)	Mean ± SD	3.05 ± 1.12	3.05–3.06
	< 2.6 mmol/L	86,113 (35.2)	35.0–35.4
Triglyceride, mmol/L (n = 282,333)	Mean ± SD	1.78 ± 1.11	1.78–1.79
	< 1.7 mmol/L	162,331 (57.5)	57.3–57.7
HDL-cholesterol, mmol/L (n = 245,335)	Mean ± SD	1.31 ± 0.47	1.30–1.31
	Men, > 1.0 mmol/L	59,874 (65.7)	65.4–66.0
	Women, > 1.3 mmol/L	69,939 (45.4)	45.1–45.6
	Overall	129,813 (52.9)	52.7–53.1
Non-HDL-cholesterol (n = 245,164)	Mean ± SD	3.84 ± 1.26	3.84–3.85
	< 3.4 mmol/L	93,028 (38.0)	37.8–38.1

Missing data—HbA1c: 4807 (1.7%); systolic blood pressure: 180 (0.1%); diastolic blood pressure: 182 (0.1%), blood pressure: 183 (0.1%); total cholesterol: 5,880 (2.0%), triglyceride: 6580 (2.3%), LDL-cholesterol: 44,271 (15.3%); HDL-cholesterol: 43,578 (15.1%).

Around 79.0% of patients had at least one type of antihypertensive drug, and ACE inhibitor (49.6%) was the most commonly used antihypertensive agent. However, over a quarter were neither on ACE inhibitors nor ARB, even though 81.6% of the cohort had hypertension. It was further observed that 2,446 (0.8%) of patients were on ACE inhibitor and ARB combination therapy. The mean BP was 135.3/78.3 mmHg, and 35.8% and 24.3% attained BP targets of < 140/80 and < 130/80 mmHg, respectively.

About 69.7% of patients were on one lipid-lowering drug, and statin was the commonest one (69.2%). The mean total cholesterol, LDL-cholesterol, HDL-cholesterol, and triglyceride were 5.13 mmol/L, 3.05 mmol/L, 1.31 mmol/L, and 1.78 mmol/L, respectively. Meanwhile, the respective achievements of LDL-cholesterol, HDL-cholesterol, triglyceride, and non-HDL-cholesterol targets were 35.2%, 52.9%, 57.5%, and 38.0%.

It was observed that 23.6% of patients were on antiplatelet therapy, and aspirin was the pharmacological agent of choice. Overall, 40.1% of our patients were on polypharmacy with five or more types of medications.

## Discussion

The demographics of our patients were consistent with the National Diabetes Registry report^6^.

Our patients were relatively younger than those reported in Singapore and Sweden registries and had an earlier age at diabetes onset^22,23^. The results are not unexpected as the National Health and Morbidity Survey in Malaysia reported a high prevalence of diabetes risk factors such as overweight/obesity, physical inactivity, hypertension, and hypercholesterolemia among the general population^10^. Some of our patients had young-onset T2D, which was associated with higher risks of mortality, macrovascular, and microvascular complications^14,23^. Previous Malaysian studies found that younger adults with T2D had poorer control of HbA1c, BP, and LDL-cholesterol^24,25^. Thus, there is a need to intensify metabolic control among this subpopulation to prevent adverse outcomes, especially after accounting for the legacy benefits of multifactorial risk interventions^26^. The higher proportion of women in our cohort can be partly explained by gender differences in attitudes, behaviours, illness orientations, willingness to seek medical care, and knowledge of diabetes^27^.

The higher proportion of abdominal obesity than obesity by BMI classification implies that more patients have apple-shaped central obesity instead of pear-shaped peripheral obesity, which is expected as abdominal obesity is closely associated with insulin resistance and T2D^28^. From the clinical management perspective, most patients would need to lose up to 10% of their weight in 6 months to improve their clinical outcomes^18^. Pharmacotherapy can be considered for those with BMI ≥ 27.0 kg/m^2^ who have failed 6 months of lifestyle modification^18^. The local CPG recommends three classes of anti-obesity agents, namely phentermine, orlistat, and liraglutide^18^. In addition, bariatric surgery may be considered for patients who fulfil specific criteria, e.g., BMI ≥ 30 kg/m^2^ with failed weight reduction attempts^18^. Although these guidelines are consistent with the International Diabetes Federation recommendations^19^, there are overt challenges to implement in real-world practice. For instance, all three classes of anti-obesity agents are not included in the MOH Medicines Formulary, which means that the drugs are not available for use in healthcare facilities under the MOH^29^. The lack of bariatric surgery training programmes and the need for patients to bear some procedure costs in public healthcare facilities further hinder adherence to these CPGs^30^.

The high prevalence of hypertension and dyslipidaemia was quite similar to those reported in Singapore^22^. The common roles of visceral obesity in the underlying pathophysiology of insulin resistance, elevated blood pressure, and dyslipidaemia may partly explain the high comorbidities^31^. Our patients had relatively low prevalences of diabetes-related complications, and the concerns about this issue among T2D patients in Malaysia had been highlighted before^8,32^. The under-screening of diabetes complications can partly account for the low figures; for example, only around 60% of patients had annual electrocardiograms and fundus examinations in 2019^6,8,32^. Besides that, the low complications can be partially due to the relatively short diabetes duration. Duration of diabetes is an independent predictor for macrovascular and microvascular complications and all-cause mortality in patients with T2D^33^. The high prevalence of multimorbidity among our patients is a concern. Multimorbidity is now a priority agenda for health policymakers and providers due to the high usage of healthcare resources^34^ and should be considered during the planning and allocating of healthcare resources in Malaysia.

The mean HbA1c of our patients was 0.37% absolute percentage points lower than the 8.34% reported in the previous nationwide survey in 2009^35^. The proportion of patients with HbA1c < 7.0% was higher than the 30.9% reported back then^35^. The results signified improved glycaemic control over the years in Malaysia. Our HbA1c performance was quite similar to the pooled global HbA1c achievement rate reported in a meta-analysis^20^. Generally, our patients underperformed compared to Europe and North American regions but outperformed the rest of the world^20^. Between Asian countries, our HbA1c performance was similar to Singapore (40.9%), outperformed The Philippines (15.0%), Bangladesh (23.1%), India (21.6%), and Saudi Arabia (20%), but underperformed when compared to Japan (44.9%) and Hong Kong, China (47%)^36–42^. These wide variations are multifactorial as various patient, physician, and healthcare system-related factors contribute to metabolic control^4,20^. Patient-related factors include their socioeconomic status, health behaviour, and compliance with the diabetes management plan, while examples of physician-related factors are their knowledge level and adherence to CPGs^20^. System-related factors include access to healthcare, medication availability, and clinical staff adequacy^20^.

Besides that, our HbA1c achievement was above the national quality standard of ≥ 30.0^7^. While these findings were laudable, a high proportion of patients had HbA1c > 8.5%, which means the Key Performance Index (KPI) target of < 20% was not met and represented a shortfall in quality of diabetes care. This is worrying because poorly controlled HbA1c is a risk factor for developing diabetes complications, which could be prevented should the excess HbA1c gap be treated to targets^18,25^.

The high utilisation of ACE inhibitors is expected because the CPGs recommend ACE inhibitor as the drug of choice to control blood pressure in patients with T2D, and ARB is the alternative when ACE inhibitor is not tolerated^18,19^. However, over a quarter of patients were neither on ACE inhibitors nor ARB, even though most had hypertension. This may signify inadequate healthcare professionals’ adherence to CPGs. Moreover, even though the CPGs recommend against the dual blockade of the renin-angiotensin system due to excessive risk of hyperkalaemia, hypotension, and renal failure^18,43^, a small percentage of our patients were on ACE inhibitor and ARB combination therapy. A systematic review reported that the healthcare professionals’ adherence to CPGs reduced after more than a year in about half of the cases^44^.

Compared with the national study in 2009, the mean systolic and diastolic BP and percentages of patients achieving the BP target were quite similar^35^, implying static progress for BP control among T2D patients in Malaysia. This highlights how difficult BP control is because almost half of our patients were on two or more antihypertensive agents. According to a meta-analysis, the global pooled achievement of BP targets was 29.0%^20^. For the specific BP goal of < 130/80 mmHg, our patients generally outperformed their European counterparts but underperformed compared to Americans and the rest of the world^20^. Between Asian countries, Malaysia performed worse than our counterparts in Japan (36.3%), Hong Kong (37%), and Thailand (28.4%)^41,42,45^. This may be partly attributed to the high proportion of central obesity among our patients, which affects blood pressure positively^31^.

The high usage of statins is expected per clinical recommendations^18,19^. Our patients’ mean total cholesterol, LDL-cholesterol, HDL-cholesterol, and triglycerides had improved compared to the previous Malaysian study^35^. The higher utilisation of lipid-lowering drugs among our patients can partly explain the improvement^35^. Among several lipid targets, the attainment of the LDL-cholesterol goal was the lowest, despite LDL-cholesterol being the sole primary lipid target.^18,19^ Between Asian countries, our performance for LDL-cholesterol was better than Japan (27.1%) but worse than Hong Kong (45%) and Thailand (41.2%)^41,42,45^. The differences could be attributed to multiple factors affecting the control of LDL-cholesterol, such as patients’ sociodemographic factors, medication compliance, and providers’ adherence to CPGs^4,20^. The global pooled target attainment rates had a similar performance sequence as in this study: triglycerides (61.9%), followed by HDL-cholesterol (58.2%) and LDL-cholesterol (49.2%)^20^. However, our patients fared worse than the global average in all lipid targets^20^. This is worrying because the gaps between actual performance and guideline recommendation represent a preventable burden of cardiovascular complications and should be optimised for better clinical and public health outcomes^46^.

The use of aspirin as the primary antiplatelet agent in our patients is expected because there are clear recommendations for aspirin in T2D patients with established cardiovascular diseases for secondary prevention of cardiovascular events^18,19^. Primary prevention of cardiovascular disease with low-dose aspirin is only recommended in patients with high cardiovascular risks and only after thorough discussion with them on the benefits versus risk of bleeding^18^.

The high proportion of patients with polypharmacy is not unexpected, considering the high multimorbidity and the need for multiple classes of treatment for metabolic control and prevention of diabetes complications^18^. Polypharmacy in diabetes management is an important issue that can lead to inappropriate drug use, under-prescription, low adherence, and side effects^47^. These, in turn, can affect metabolic control and the prevention of complications over a longer term^47^.

This study has several strengths. Our paper describes the formation of a 10-year retrospective cohort dataset that may serve as guidance so that similar cohort datasets can be integrated from the abundance of pre-existing cross-sectional administrative datasets. Our dataset benefits from a large sample size of real-world clinical data that opens opportunities to answer research questions requiring longitudinal data analysis. The analysis of treatment profiles and metabolic control reflects the quality of diabetes care in Malaysia, which has generally improved over the years^35^. Nevertheless, we highlighted some inadequate adherence to CPGs, the shortfall in the HbA1c Key Performance Index, and inadequate improvement in blood pressure and cholesterol control. These findings may catalyse data-driven decision-making, and we recommend clinicians, diabetes programme managers, and health policymakers to consider these issues in the multidisciplinary team approach to increase the quality of T2D care in Malaysia.

There are also some limitations to this study. Firstly, random error during clinical audits could occur when data was wrongly transcribed into the web-based registry. Nevertheless, the random error would be mitigated by the large sample size^9^. Clinical examinations that were not done or recorded and data that were not transcribed would cause missing data, which were relatively low for most clinical variables, except for waist circumference/abdominal obesity, LDL-cholesterol, and HDL-cholesterol. Since the audit process was manual and medical records in most MOH health clinics were paper-based, universal data entry for clinical variables was not feasible and was an acknowledged limitation of the registry^6^. In this descriptive study, we reported based on complete case analyses, which reflect the real-world clinical scenario. Finally, some essential information, such as socioeconomic factors, hyperglycaemic or hypoglycaemic crises, and insulin types, are unavailable in the National Diabetes Registry. These data are recommended by the Lancet Commission on Diabetes to establish a diabetes register for risk stratification, clinical management, and monitoring purposes^4^.

## Conclusion

HbA1c, blood pressure, and lipids were not optimally controlled among 288,913 T2D patients in Malaysia. The study findings can be capitalized as a guideline by clinicians, programme managers, and health policymakers to improve the quality of diabetes care and prevent long-term complications. We recommend further research on diabetes policy, cost analysis, and qualitative exploration of perspectives, practices, and barriers to self-care diabetes practices to understand the diabetes epidemic in Malaysia better.

## Data Availability

The National Diabetes Registry dataset retrieved and analysed in this study is not available publicly due to local ethics regulation and could be obtained via written permissions to the Director General of Health, Malaysia.
